# Dose Dependent Activation of Retinoic Acid-Inducible Gene-I Promotes Both Proliferation and Apoptosis Signals in Human Head and Neck Squamous Cell Carcinoma

**DOI:** 10.1371/journal.pone.0058273

**Published:** 2013-03-04

**Authors:** Jingzhou Hu, Yue He, Ming Yan, Chao Zhu, Weimin Ye, Hanguang Zhu, Wantao Chen, Chenping Zhang, Zhiyuan Zhang

**Affiliations:** 1 Department of Oral & Maxillofacial-Head & Neck Oncology, Shanghai Ninth People’s Hospital, Shanghai Jiao Tong University School of Medicine, Shanghai, China; 2 Shanghai Key Laboratory of Stomatology, Shanghai Ninth People’s Hospital, Shanghai Jiao Tong University School of Medicine, Shanghai, China; University of Missouri-Columbia, United States of America

## Abstract

The retinoic-acid-inducible gene (RIG)-like receptor (RLR) family proteins are major pathogen reorganization receptors (PRR) responsible for detection of viral RNA, which initiates antiviral response. Here, we evaluated the functional role of one RLR family member, RIG-I, in human head and neck squamous cell carcinoma (HNSCC). RIG-I is abundantly expressed both in poorly-differentiated primary cancer and lymph node metastasis, but not in normal adjacent tissues. Activation of RIG-I by transfection with low dose of 5′-triphosphate RNA (3p-RNA) induces low levels of interferon and proinflammatory cytokines and promotes NF-κB- and Akt-dependent cell proliferation, migration and invasion. In contrast, activation of RIG-I by a high dose of 3p-RNA induces robust mitochondria-derived apoptosis accompanied by decreased activation of Akt, which is independent of the interferon and TNFα receptor, but can be rescued by over-expression of constitutively active Akt. Furthermore, co-immunoprecipitation experiments indicate that the CARD domain of RIG-I is essential for inducing apoptosis by interacting with caspase-9. Together, our results reveal a dual role of RIG-I in HNSCC through regulating activation of Akt, in which RIG-I activation by low-dose viral dsRNA increases host cell surviral, whereas higher level of RIG-I activation leads to apopotosis. These findings highlight the therapeutic potential of dsRNA mediated RIG-I activation in the treatment of HNSCC.

## Introduction

The cellular innate immune response is the first line of host defense against viruses and other pathogens. As host cells, cancer cells and virus-infected cells share certain properties, such as the expression of specific antigens and the need to evade immune and non-immune control mechanisms in order to persist [1], [2]. To suppress viral replication and spread, host cells often undergo premature cell death by triggering apoptosis. Apoptosis is therefore considered a potent antiviral defense mechanism by which infected cells are eliminated from the host. Tumor cells could be more susceptible to this kind of death signal than nonmalignant cells, many alterations required for tumor formation can also result in increased vulnerability to certain apoptotic stimuli [3]. Therefore, triggering anti-viral responses may be applied as an effective tumor therapy approach.

Double stranded RNA (dsRNA), generated during infection with both RNA and DNA viruses, is a strong inducer of host antiviral responses. Mammals have several families of pattern recognition receptors (PRRs), e.g., Toll-like receptors (TLRs), Retinoic acid-inducible gene (RIG) like receptors (RLRs), and Nod-like receptors (NLRs), to recognize viral dsRNA [4], [5]. The innate immune responses to virus infection are often initiated by Toll-like receptors; alternatively, cytoplasmic dsRNA-recognizing RNA helicases RIG-I and melanoma differentiation–associated antigen 5 (MDA-5) can initiate antiviral signaling [5]. RIG-I is localized in the cytosol and recognizes 5′-triphosphate RNA (3p-RNA) generated by viral RNA polymerases in the cytosol of cells [6], [7]. Polyinosinic-polycytidylic acid [poly(I:C)], a synthetic and artificial mimic of long double-stranded RNA, is a strong activator of MDA-5. Upon recognition of RNA ligands, RIG-I and MDA-5 bind to the adapter protein interferon-β (IFN-β) promoter stimulator 1 (IPS-1) (also known as CARDIF, MAVS or VISA) located in the outer mitochondrial membrane [8], [9]. The interactions of RIG-I or MDA-5 with IPS-1 initiate signaling pathways that elicit the activation of transcription factors including IFN regulatory factor 3 (IRF-3) and nuclear factor-κB (NF-κB), resulting in IFN production, activation of NF-κB target genes and the secondary induction of IFN-stimulated genes [5], [10].

On the other hand, apoptosis has been known one of the important RLR activation-mediated antiviral responses in many cells. However, apoptotic mechanisms triggered by different virus in different cells appeared to be quite complex. For instance, it has been reported that the activation of RIG-I and MDA-5 using 3p-RNA and poly(I:C) leads to apoptosis of human melanoma cells, which proved to be independent of type I IFNs but dependent on upregulation of Puma and Noxa [11], while RIG-I-mediated activation of IRF-3 was demonstrated to be required for the apoptotic effect of adenovirus infection in the fibrosarcoma cells [12], [13]. Head and neck squamous cell carcinoma (HNSCC) is the sixth most common cancer worldwide, affecting 600,000 new patients each year. In the United States, 50,000 new cases are diagnosed, and nearly 10,000 deaths are attributable to this disease annually [14]. Despite advances in multimodality therapy, the overall 5-year survival rate is 40–50%, and has increased only incrementally in the past two decades [15], [16]. Although RLR activation-mediated apoptosis is a potentially effective approach to tumor therapy, whether it also leads to apoptosis in HNSCC cells and/or the molecular mechanisms involved still remain largerly elusive. Developing new therapeutics targeting signaling molecules contributing to the evasion of apoptosis in HNSCC may effectively render those cancer cells susceptible to natural or induced programmed cell death.

To investigate whether RLRs also function in HNSCC, here we first examined the expression of RIG-I and MDA5 in both tumor biopsies and HNSCC cell lines. Then, we tested the impact of RLR activation by transfection with various doses of 3p-RNA or poly (I:C) on cell proliferation, migration and production of proinflammatory cytokines and type I interferon in above cell lines. Our results showed that activation of RLR with low dose of 3p-RNA promoted cell proliferation, whereas that with a high dose of 3p-RNA led to robust apoptosis in HNSCC cells. Furthermore, we demonstrated that the dual role of RIG-I in regulating the activation of Akt in proliferation and apoptosis.

## Materials and Methods

### Ethics Statement

This study was approved by the Ethics Committee of Shanghai Ninth People’s Hospital, Shanghai Jiao Tong University School of Medicine and carried out according to the recommendations of the Declaration of Helsinki. No informed consent (written or verbal) was obtained for use of retrospective tissue samples from the patients within this study, some of whom were deceased, since the Ethics Committee, who waived the need for consent, did not deem this necessary. All samples were anonymous.

### Reagents, Plasmids Constructs and siRNA

3p-RNA and blunt-end dsRNA (BE-RNA) were obtained from Invitrogen (Carlsbad, CA, USA). M-PER® Mammalian Protein Extraction Reagent, NE-PER Nuclear and Cytoplasmic Extraction Reagents, BCA™ Protein Assay Kit and SuperSignal West Femto Maximum Sensitivity Substrate were purchased from Pierce (Rockford, IL, USA). Polyclonal antibodies against lamin A, monoclonal antibody for MDA-5, caspase-8 p20, caspase-3, caspase-9, cleaved caspase-9 (Asp330), PARP, MMP-9 (G657), PP2A C subunit, PTEN, NF-κB p65, IRF3, extracellular signal regulated kinase p44/p42 (ERK1/2, Thr202/Tyr204), c-Jun N-terminal kinase/stress associated protein kinase (JNK/SAPK, Thr183/Tyr185), p38 (Thr180/Tyr182), phosphor-Akt (Ser473), phosphor-Akt (Thr308), phosphor-p85 (Tyr458), I-κB, phosphor-IKKα/β (Ser176/180) and β-actin were from Cell Signaling (Beverly, MA, USA). The antibody against RIG-I was from Abcam (Cambridge, MA, USA). Chemical inhibitors PDTC (10 µM), wortmannin (25 µM), PD98059 (10 µM), SB203580 (10 µM) and SP600125 (10 µM) were from Calbiochem (Darmstadt, Germany). The recombinant vectors encoding RIG-I (NM_024119), Akt (NM_005163) and their mutants constructed by polymerase chain reaction (PCR)-based amplification from CAL27 or SCC4 cDNA were subcloned into the pcDNA3.1 eukaryotic expression vector (Invitrogen, San Diego, CA, USA). Constitutively active Akt was prepared as previously reported [17]. Small interfering RNA (siRNA) targeting RIG-I and the control duplex, anti c-Jun/AP-1(sc-44) were from Santa Cruz Biotechnology (Santa Cruz, CA, USA). For transient transfection of plasmids or siRNA in CAL27 or SCC4 cells, the jetPEI and INTERFER reagents were used, respectively (Polyplus, Illkirch, France).

### Cell Culture

Human head and neck squamous cell carcinoma SCC4, SCC9, SCC25 and CAL27 cells were purchased from American Type Culture Collection (Manassas, VA, USA) and were cultured as previously reported [18]. The cells were maintained in Dulbecco’s modified Eagle’s medium (DMEM, GIBCO BRL, Grand Island, NY, USA) supplemented with 10% heat-inactivated fetal bovine serum (FBS, GIBCO BRL), penicillin (100 U/mL) and streptomycin (100 µg/mL). They were incubated at 37°C in humidified atmosphere containing 5% CO_2_.

### Immunohistochemistry and mRNA Analysis of RIG-I Expression in HNSCC Tissues

Human HNSCC tissues for immunohistochemistry and mRNA expression were obtained from the tissue library of Shanghai Ninth People’s Hospital, Shanghai Jiao Tong University School of Medicine. Sixty-one samples of 34 patients with primary HNSCC who underwent surgery in our department from January 2008 to December 2011 were recruited in this study. The tissues prepared for immunohistochemistry were fixed with formalin, embedded in paraffin and immunostained with an anti-RIG-I antibody using the avidin-biotin peroxidase complex method. The intensity of staining seen were analyzed according to criteria described by Malik et al. [19]. The intensity is designated as negative when no tumor cells stain, weak staining when 10–20% of cells stain, moderate staining when 20–50% of cells stain, and strong staining when over 50% of cells stain. Tissues prepared for mRNA expression were immediately frozen in isopentane cooled by liquid nitrogen and stored until use at −80°C. Each tissue was pathologically confirmed (graded as 1 to 3 according to the WHO standard classification of HNSCC, 2005 version).

### RNA Quantification

Quantitative real-time RT-PCR (Q-PCR) analysis was performed using a LightCycler (Roche, Basel, Switzerland) and SYBR RT-PCR kit (Takara, Dalian, China). Data were normalized to β-actin expression.

### Assays for Cell Proliferation, Migration and Invasion

For 3-(4,5-dimethylthiazol-2-yl)-2,5-diphenyltetrazolium bromide (MTT) assays, CAL27 cells or SCC4 cells (2,000 per well) were seeded in 96-well plates, cultured for 12 h, serum starved overnight and then transfected with 3p-RNA or BE-RNA for 16 h. Cell growth was assessed by the MTT assay (Promega, Madison, WI, USA), as described by the manufacturer. Each experiment consisted of three to four replicate wells for each treatment dose, and each experiment was performed at least three times. *In vitro* migration and invasion assays were performed using Matrigel coated and uncoated 24-well chambers/microfilters (5 µm pore polycarbonate filters), respectively, according to the manufacturer’s instructions (BD Bioscience, Bedford, MA). Brieﬂy, after rehydration of the chambers, the CAL27 cells were transfected with 100 ng/ml 3p-RNA for 16 h or BE-RNA, and then the cells were harvested and seeded in 200 µl of RPMI1640 plus 5% FBS onto the upper chamber (1×10^5^ cells per chamber), while 600 µl of RPMI1640 plus 10% FBS was placed in the lower chamber. After 24 h, CAL27 cells that had migrated to the underside of the membrane were stained using Leucocrystal Violet. The number of cells in the membrane was counted in 20 randomly selected field views under a 100× objective.

### Apoptosis Assay

Cells were washed, resuspended in the staining buffer and examined with the Vybrant Apoptosis kit (Invitrogen) according to the manufacturer’s instructions. Cells were stained with Rhodamine123 (Molecular Probes, 0.1 µg/ml) for 15 min before collection and then analyzed by fluorescence-activated cell sorting (FACScalibur, Becton Dickinson, Mountain View, CA, USA).

### Western Blotting and Immunoprecipitation

After disrupting cells in lysis buffer, the cellular proteins were loaded and separated on SDS–PAGE and transferred to a nitrocellulose membrane (Amersham Biosciences, Piscataway, NJ, USA) using a standard electric transfer protocol. The membrane was blocked, probed with primary antibodies and then incubated with a horse radish peroxidase (HRP)-labeled secondary antibody (DAKO, Carpinteria, CA, USA). The protein signals were developed for visualization with the TMB Membrane Peroxidase Substrate (KPL, Gaithersburg, MD, USA).

For immunoprecipitation, CAL27 or SCC4 cell lysates were incubated with anti-RIG-I or anti-caspase-9 antibody cross-linked to protein A-Sepharose beads and prepared for analysis as previously described [11].

### Statistical Analysis

Data are presented as means ± standard deviation (S.D.) of more than three independent experiments. Statistical analysis was performed using the Chi-square test (only for biopsies) and Student’s t-test. *P* values less than 0.05 were considered to be significant.

## Results

### RIG-I Expression in HNSCC Biopsies and Cell Lines

**Figure 1:**
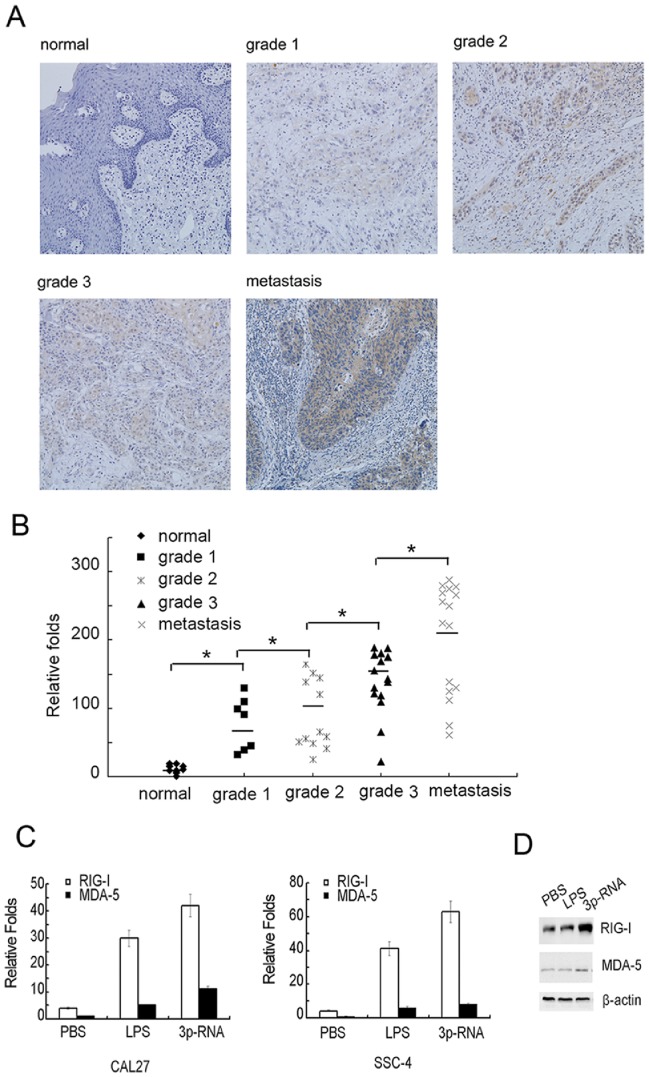
RIG-I is highly expressed on HNSCC cells. (A) Representative results of immunohistochemical staining of RIG-I protein (yellow) in HNSCC tissue samples. Photos were taken under 200× magnification. (B) mRNA expression of RIG-I in HNSCC tissue samples same to Table 1 were analyzed by Q-PCR, the lowest expression of RIG-I in normal control sample as control (*, *P<*0.001, n = 61, ANOVA). (C) CAL27 or SCC4 cells (5×10^5^ per well) were plated in 12-well plates overnight and then stimulated with PBS or LPS for 12 h or transfected with 100 ng/ml 3p-RNA for 16 h. mRNA expression levels of RIG-I and MDA-5 were analyzed by Q-PCR. The basal level of MDA-5 normalized by β-actin was used as the control. (D) CAL27 cells (2×10^6^ per well) were plated in 6-well plates and stimulated as in (C). Protein expression levels of RIG-I and MDA-5 were determined by Western blot with β-actin as the loading control.

To evaluate RIG-I expression in HNSCC, we first assayed the protein level in biopsy samples (Table 1). RIG-I was found to be substantially expressed in a high percentage of HNSCC tissues (82.4%, 28/34), whereas it was not detected in any of the adjacent normal tissues tested (0%, 0/11). Moreover, increased staining intensity of RIG-I was associated with the pathological grade of HNSCC. While none of the well-differentiated primary tumors (grade 1) and only 41% of moderately differentiated carcinoma cells (grade 2) exhibited strong staining intensities; however, about 80% of the poorly differentiated (grade 3) and 87% of lymph node metastatic tumors exhibited strong staining for RIG-I (*P<0.01, Chi-square test). Typical staining patterns are shown in Figure 1A. However, MDA5 was found to be expressed weakly in all primary tumor tissues (data not shown). To further confirm the relationship between expression of RIG-I and progress of HNSCC tumor progression, we detected the mRNA expression in HNSCC biopsy samples from Table 1 by Q-PCR. Our results indicated that the expression of RIG-I mRNA was also correlated with the progression of HNSCC (Fig. 1B). In addition, we also assayed the mRNA expression level in the HNSCC cell lines SCC4, CAL27 (Fig. 1C), SCC9 and SCC25 (Figure S1), in which the basal expression level of RIG-I was much higher than that of MDA-5, and RIG-I was more potently induced than MDA-5 upon stimulation with the TLR4 ligand lipopolysaccharide (LPS), or transfection with 3p-RNA. Finally, we further confirmed the inducible expression of RIG-I at the protein level in CAL27 cells (Fig. 1D). Together, all these results revealed a positive correlation of RIG-I expression with development and progression of HNSCC.

**Table 1:** Relationship between RIG-I staining intensity versus severity of HNSCC.

Pathologicgrade	n	Negative	Weak staining	Strong staining	Positive
Adjacent normal tissue	11	11	0	0	0
Grade 1	7	3	4	0	4
Grade 2	12	1	6	5	11
Grade 3	15	2	1	12	13
Lymph node metastasis	16	0	2	14	16

### RIG-I Activation by Low dose 3p-RNA Leads to Increased Cell Proliferation, Migration and Invasion

**Figure 2:**
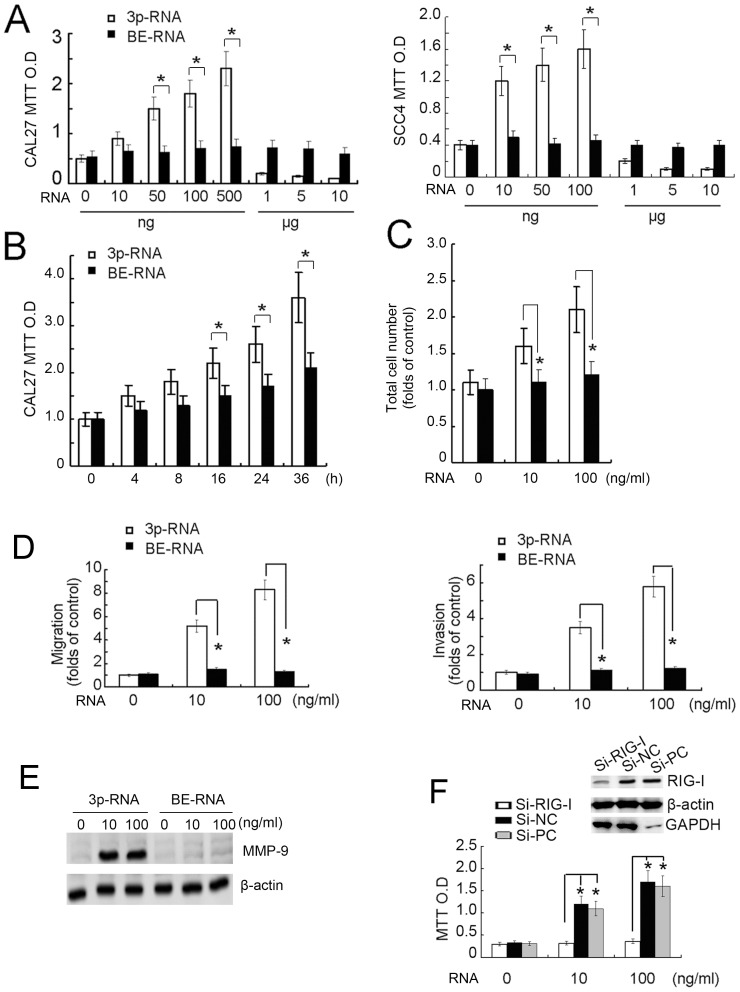
Transfection of low-dose 3p-RNA leads to increased cell proliferation. (A) CAL27 or SCC4 cells were pre-transfected with various dose of 3p-RNA as indicated for 16 h before the MTT absorption assay with BE-RNA as a control. (B) CAL27 cells were pre-transfected with various dose of 3p-RNA (100 ng/ml) for indicated time period before the MTT absorption assay with BE-RNA as a control. (C) CAL27 cells were pre-transfected with various dose of 3p-RNA as indicated for 36 h before cell counting by FACS with BE-RNA as a control. (D) CAL27 cells treated as in A were collected for migration and invasion assays. Results are presented as fold increases over the basal level of the control. The numbers of cells in the membranes were analyzed after 24 h. (E) CAL27 cells (2×10^6^ per well) were cultured in 6-well plates and transfected with indicated doses of 3p-RNA or BE-RNA for 24 h. Cell lysates were collected and analyzed by Western blot using the indicated antibodies. (F) CAL27 cells were pre-transfected with RIG-I-specific siRNA, negative control (Si-NC) or GAPDH-specific positive control (Si-PC) for 36 h before transfection with the indicated dose of 3p-RNA for 16 h. Cell lysates were also collected for monitoring knockdown efficiency. MTT results are presented as the absorption radio. Similar results were obtained in multiple repetitions (at least three) of these experiments (A, B, C, D, F, *P<0.01, Student’s t-test).

Given the predominant expression of RIG-I compared with MDA-5 in HNSCC, we then focused our study on the functional role of RIG-I in HNSCC. As shown in Figure 2A, we found that transfection of 3p-RNA into both CAL27 and SCC4 cells at doses between 10–500 ng/ml induced cell proliferation when compared with the BE-RNA control. However, the number of live cells transfected with high doses (1–10 µg/ml) of 3p-RNA were significantly decreased (Fig. 2A). Low dose (100 ng/ml) of 3p-RNA transfection promoted CAL27 cell proliferation in time dependent manner (Fig. 2B). Furthermore, low dose of 3p-RNA transfection increased CAL27 cell number (Fig. 2C), which further indicates the pro-proliferative function of 3p-RNA. In addition, low doses of 3p-RNA also promoted cell migration and invasion of CAL27 (Fig. 2D) and SCC4 (Figure S2) cells, and stimulated MMP-9 expression (Fig. 2E). Finally, siRNA knock-down of RIG-1 in CAL27 cells inhibited low-dose 3p-RNA-mediated cell proliferation (Fig. 2F), suggesting that low-dose 3p-RNA induced activation of RIG-I promotes proliferation, migration and invasion of HNSCC cells.

### Activation of RIG-I by Low-dose 3p-RNA Induces Low Levels of Proinflammatory Cytokines and Type I Interferon

**Figure 3:**
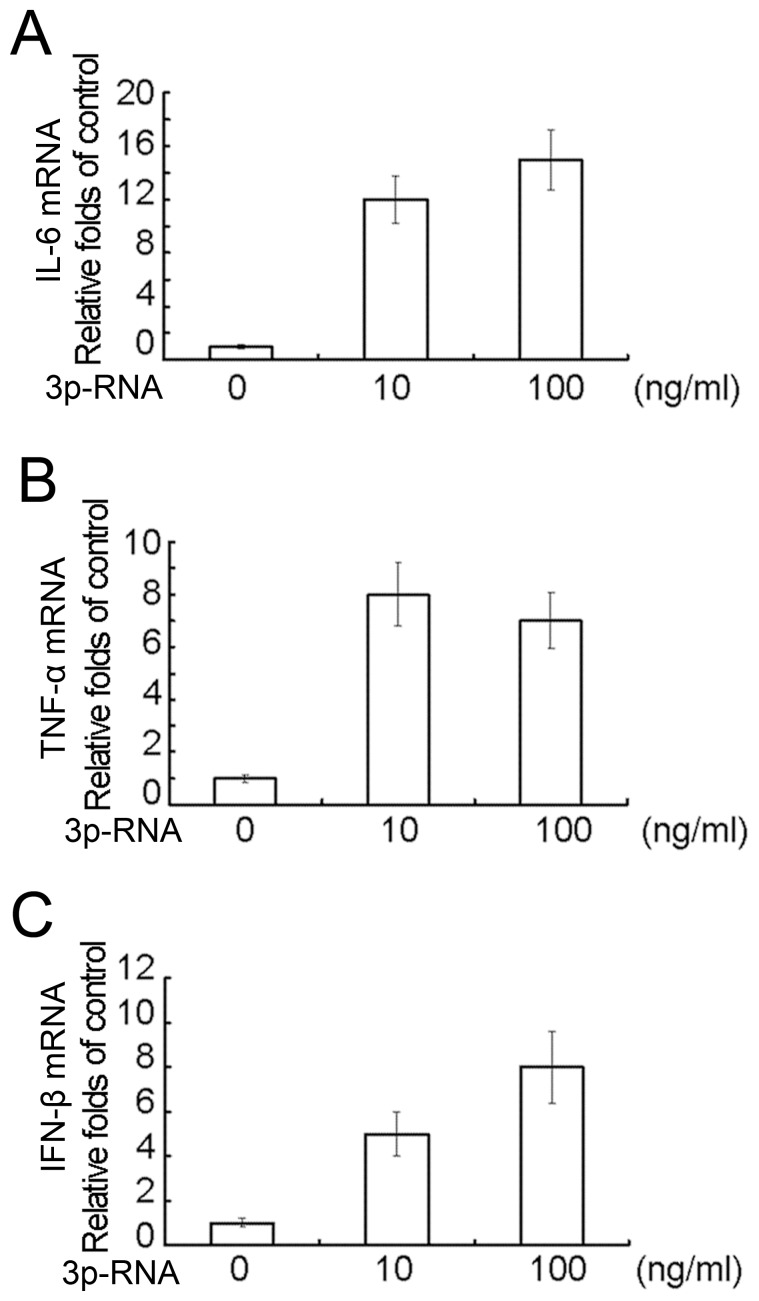
Transfection of low-dose 3p-RNA induces low levels of proinflammatory cytokines and type I interferon. CAL27 cells (5×10^5^ per well) were cultured in 12-well plates overnight and then transfected with indicate doses of 3p-RNA for 16 h with BE-RNA as a control. Expression of IL-6(A), TNFα(B) and IFN-β(C) was assayed by Q-PCR. Similar results were obtained in multiple repetitions (at least three) of these experiments.

Given that RIG-I functions as a major virus RNA PRR, the effects of low-dose 3p-RNA on levels of type I interferon and proinflammatory cytokines in CAL27 cells were then assayed by Q-PCR. The mRNA expression levels of TNFα (Fig. 3A), IL-6 (Fig. 3B) and interferon β (Fig. 3C) were all weakly induced [12 to 30-fold of the unstimulated basal level compared with over 200-fold induction in macrophages (data not shown)]. We further assayed the function of IFN-β and TNFα on cell proliferation by knockdown of IFNRα and TNFR1 simultaneously. Knockdown of these two receptors did not affect the low-dose 3p-RNA-induced cell proliferation by MTT assay (data not shown), which may result from very low dose of IFN-β and TNFα induced by 3p-RNA transfection. These results indicate that activation of RIG-I by low-dose 3p-RNA induces low levels of proinflammatory cytokines and type I interferon in HNSCC cells.

### Activation of RIG-I by Low-dose 3p-RNA Induces Activation of NF-κB, MAPK and Akt

**Figure 4:**
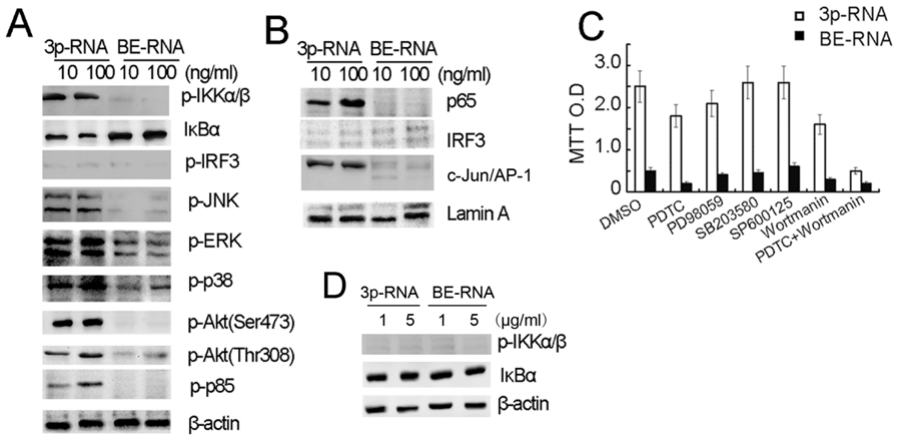
Transfection with low-dose 3p-RNA induces activation of NF-κB, MAPK and Akt. (A) CAL27 cells (2×10^6^ per well) were cultured in 6-well plates and transfected with indicated doses of 3p-RNA or BE-RNA for 16 h. Cell lysates were collected and analyzed by Western blot using the indicated antibodies. (B) Nuclear extracts prepared from CAL27 cells transfected as in (A) were subjected to Western blot analysis with anti-NF-κB p65, IRF3 and AP-1 antibodies. (C) CAL27 cells transfected as in (A) were treated with indicated inhibitors for 12 h and then collected for the MTT assay. (D) CAL27 cells (2×10^6^ per well) were cultured in 6-well plates and transfected with indicated doses of 3p-RNA or BE-RNA for 16 h. Cell lysates were collected and analyzed by Western blot using the indicated antibodies. Results are presented as the absorption ratio. Similar results were obtained in multiple repetitions (at least three) of these experiments.

We next investigated the molecular signaling downstream of RIG-I activation that promotes cell proliferation of CAL27 cells. As shown in Figure 4A, transfection with a low dose of 3p-RNA strongly induced NF-κB and MAPK signaling pathways, while it had a modest effect on IRF3 activation. Consistent with this, low dose of 3p-RNA greatly induced nuclear translocation of p65, c-Jun/AP1 and IRF3 (Fig. 4B). In further support of pro-proliferation role of low-dose 3p-RNA, our results also showed that low dose of transfected 3p-RNA also strongly induced phosphorylation of Akt and p85 in the PI3K pathway (Fig. 4A).

To determine which above-mentioned signaling pathways are responsible for the induction of proliferation by low-dose 3p-RNA, we treated CAL27 cells with pharmacological inhibitors of NF-κB, MAPK and PI3K (upstream of Akt) (Fig. 4C). Individual inhibitors could only partially block the proliferation, while the combination of NF-κB and Akt inhibitors decreased the proliferation to a basal level. However, high dose of 3p-RNA transfection could barely activate NF-κB (Fig. 4D).The results indicate that the proliferation induced via RIG-I activation by transfection with low-dose 3p-RNA is dependent on both NF-κB and Akt activation.

### Activation of RIG-I by High-dose 3p-RNA Induces Robust Apoptosis

**Figure 5:**
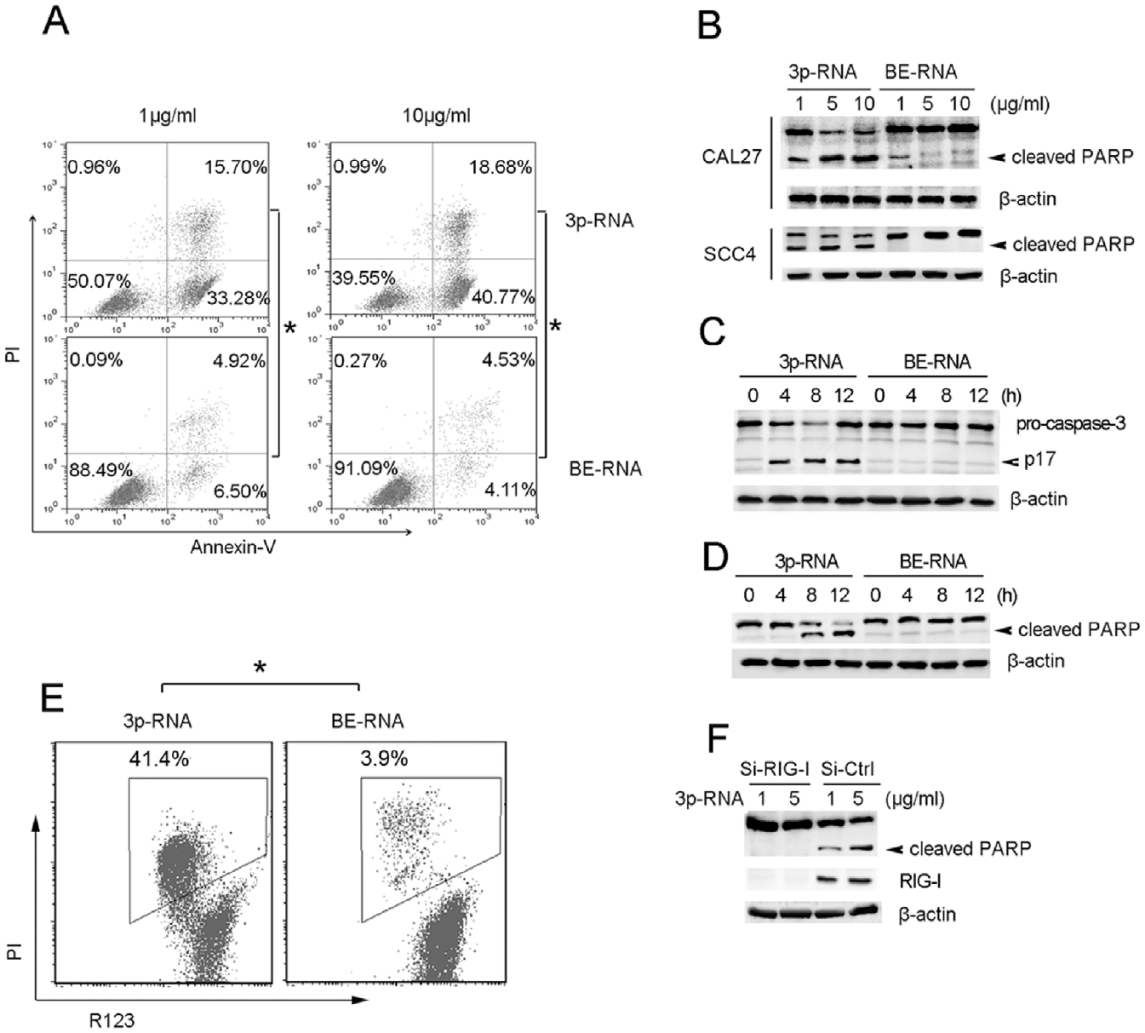
Transfection of high-dose 3p-RNA induces robust apoptosis. (A) CAL27 cells were transfected with the indicated dose of 3p-RNA and BE-RNA at corresponding doses as the control for 16 h and then stained with Annexin V and propidium iodide. Apoptotic cells (Annexin V positive cells) are indicated as the percentage of gated cells. (B) Lysates of CAL27 or SCC4 cells transfected with indicated doses of 3p-RNA or control for 16 h were immunoblotted with a PARP antibody. (C) Lysates of CAL27 or SCC4 cells transfected with 5 µg/ml of 3p-RNA or control for the indicated times were immunoblotted with a caspase-3 antibody. (D) Lysates of CAL27 cells transfected with 5 µg/ml of 3p-RNA or control for the indicated times were immunoblotted with a PARP antibody. (E) CAL27 cells transfected with 3p-RNA (5 µg/ml) or BE-RNA (5 µg/ml) for 4 h were stained with Rodamine 123 and propidium iodide. Apoptotic cells (R123 negative cells) are indicated as the percentage of gated cells. (F) CAL27 cells were pre-transfected with RIG-I-specific siRNA or negative control (si-Mock) 36 h before transfection with indicated doses of 3p-RNA for 16 h. Cell lysates were assayed by Western blot using a PARP antibody. β-actin was used as the loading control in (B), (C), (D), (F). Similar results were obtained in multiple repetitions (at least three) of these experiments.

As it was found above that high doses (1–10 µg/ml) of 3p-RNA reduced the number of viable cells (Fig. 2A), we wanted to confirm whether apoptosis is involved. Transfection with high doses of 3p-RNA (1, 10 µg/ml) led to high levels of apoptosis in both CAL27 (Fig. 5A) and SCC4 (Figure S3) cells. The cleavage of poly (ADPribose) polymerase (PARP), a prominent marker of apoptosis, was greatly induced by 3p-RNA at the concentration of 5 µg/ml, when compared to at 1 µg/ml in both CAL27 and SCC4 cells (Fig. 5B), while low dose of 3p-RNA did not induce PARP cleavage (data not shown). The induction of apoptosis by high concentrations of transfected 3p-RNA was also confirmed by activation of caspase-3 (Fig. 5C). At the same time, we found that high-dose 3p-RNA (5 µg/ml) potently induced cleavage of PARP even 8 h after transfection (Fig. 5D), and that the mitochondrial transmenbrane potential (ΔΨ m) was also damaged as early as 4 h after transfection, indicating that a high dose of 3p-RNA transfection led to mitochondria-derived apoptosis (Fig. 5E). The dependence on RIG-I of apoptosis induced by high-dose 3p-RNA was further confirmed by siRNA mediated inhibition of RIG-I (Fig. 5F). RIG-I knockdown nearly abrogated the 3p-RNA induced cleavage of PARP. Consistent with a previous report [11], knockdown of IFNRα did not affect the cleavage of PARP (data not shown). In addition, knockdown of IFNRα and TNFR1 simultaneously did not affect the high dose of 3p-RNA-induced PARP activation (data not shown), which may results from very low dose of IFN-β and TNFα induced by 3p-RNA transfection. These results indicate that the high dose of transfected 3p-RNA led to robust apoptosis in HNSCC cells, likely derived from the mitochondrial pathway.

### Transfection of High-dose 3p-RNA Leads to Mitochondria-derived Apoptosis

**Figure 6:**
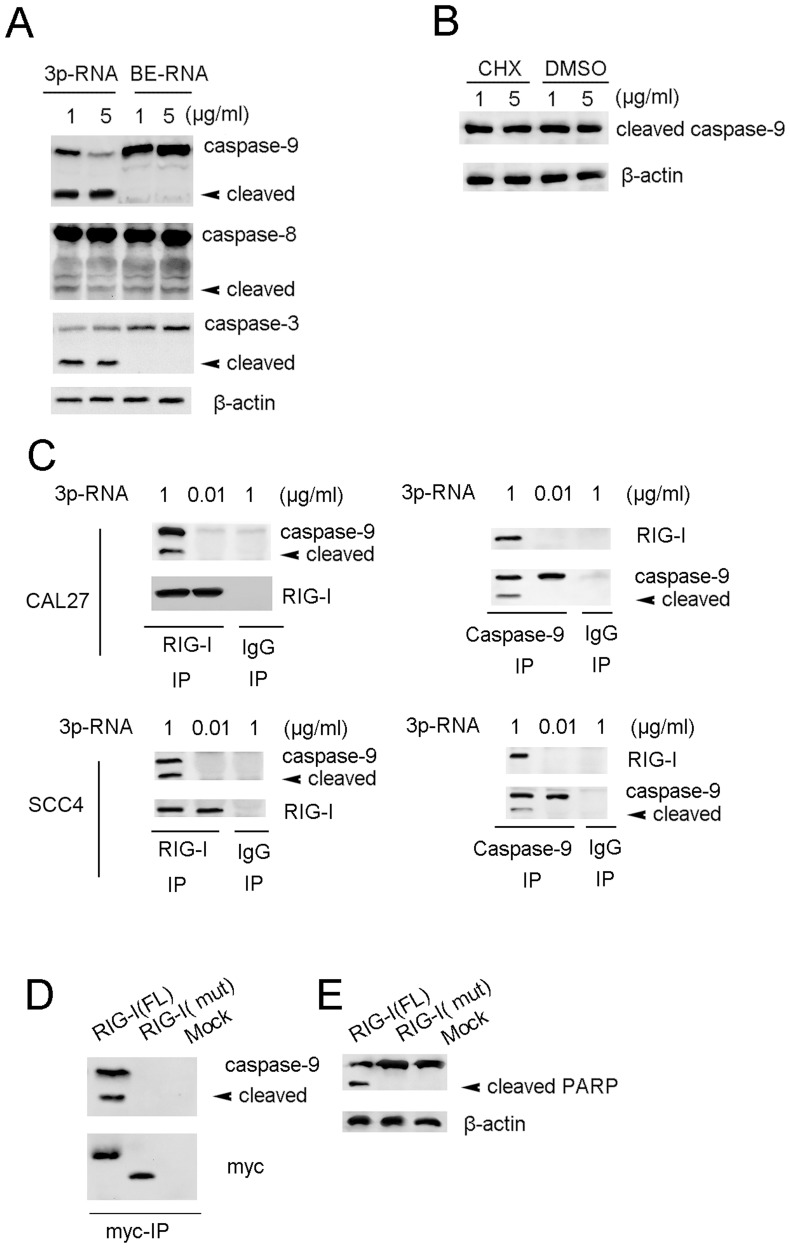
Transfection of high-dose 3p-RNA leads to mitochondria-derived apoptosis. (A) Lysates of CA27 cells transfected with indicated doses of 3p-RNA or BE-RNA for 16 h were immunoblotted with indicated antibodies with β-actin as a loading control. (B) Lysates of CA27 cells pre-transfected with indicated doses of 3p-RNA for 2 h were treated with CHX (5 µg/ml) for 12 h and then immunoblotted with a caspase-9 antibody, and β-actin was detected as a loading control. (C) Lysates of CAL27 or SCC4 cells transfected with indicated doses of 3p-RNA for 16 h were immunoprecipitated with RIG-I or caspase-9 antibodies and immunoblotted with indicated antibodies. (D) Lysates of CAL27 cells pre-transfected with myc-tagged full-length (RIG-I-FL) RIG-I, CARD domain depleted RIG-I (RIGI-I-mut) or mock plasmids for 24 h were immunoprecipitated with an anti-myc antibody, followed by immunoblotting with a caspase-9 antibody. (E) Lysates from (D) were immunoblotted with a PARP antibody with β-actin as a loading control. Similar results were obtained in multiple repetitions (at least three) of these experiments.

To investigate the apoptotic mechanisms, induced by high-dose 3p-RNA, we next examined the activation of caspase-8, caspase-9 and caspase-3. Transfection of high-dose 3p-RNA induced strong activation of caspase-9, but weak activation of caspase-8 (Fig. 6A), indicating that the high dose of 3p-RNA led to mitochondria-derived apoptosis. The activation of caspase-9 could not be inhibited by the protein synthesis inhibitor cycloheximide (CHX) (Fig. 6B), suggesting that the 3p-RNA induced apoptosis is not dependent on new protein synthesis. By immunoprecipitating endogenous RIG-I, we further found that transfection with high-dose 3p-RNA led to the interaction of RIG-I with both pro-caspase-9 and cleaved caspase-9, while a low dose of transfected 3p-RNA did not. As the antibody for total caspase-9 was not suitable for immunoprecipitation, we used a cleaved caspase-9 (Asp330) antibody in the reciprocal immunoprecipitation. Cleaved caspase-9 could also interact with RIG-I transfected with a high dose of 3p-RNA, but not in those transfected with a low dose of 3p-RNA in both CAL27 and SCC4 cells (Fig. 6C). We further overexpressed the myc-tagged full-length or CARD domain-depleted mutant of RIG-I in HEK293 cells. The CARD domain-depleted RIG-I mutant could not interact with caspase-9 (Fig. 6D). Overexpression of the CARD domain-depleted RIG-I mutant also blocked 3p-RNA induced PARP cleavage in CAL27 cells (Fig. 6E). Taken together, these results demonstrate that the high dose of transfected 3p-RNA induced apoptosis by promoting interaction of RIG-I with caspase-9.

### Apoptosis Induced by High-dose 3p-RNA can be Rescued by Constitutively Active Akt

**Figure 7:**
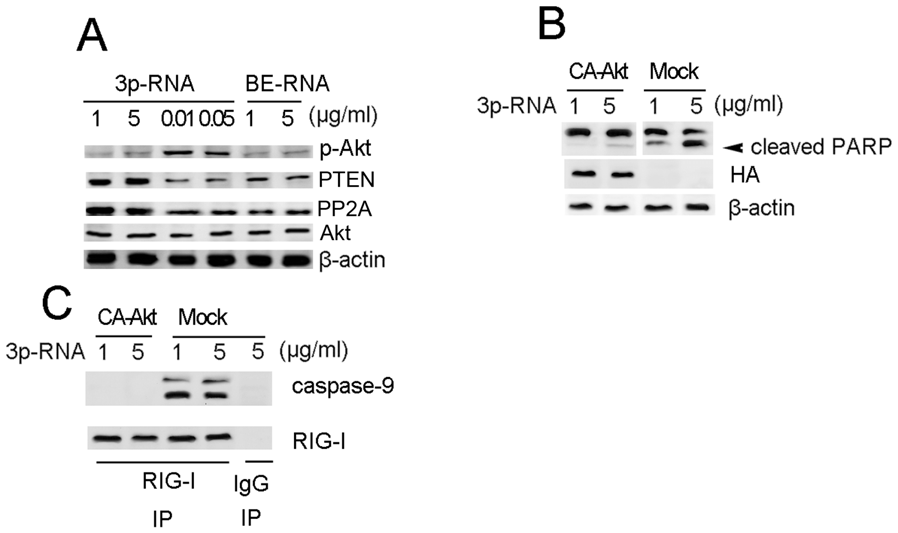
Apoptosis induced by transfection with high-dose 3p-RNA can be rescued by constitutively-active Akt. (A) Lysates of CAL27 cells transfected with indicated doses of 3p-RNA or BE-RNA for 16 h were immunoblotted with indicated antibodies with total Akt and β-actin as a loading control. (B) Lysates of CAL27 cells pre-transfected with HA-tagged constitutively-active Akt or empty plasmid (mock) for 24 h and then transfected with indicated doses of 3p-RNA for 16 h were immunoblotted with PARP or HA antibody with β-actin as a loading control. (C) Cell lysates from (B) were immunoprecipitated with a RIG-I antibody and immunoblotted with a caspase-9 antibody. Similar results were obtained in multiple repetitions (at least three) of these experiments.

To understand why 3p-RNA concentration-dependent activation of RIG-I can lead to both proliferation and apoptosis, we examined the Akt activation. While a high dose of transfected 3p-RNA induced weaker Akt activation, it led to a higher expression of Akt phosphatase PTEN and PP2A than did the low dose of 3p-RNA (Fig. 7A). To further demonstrate the role of Akt activation, we overexpressed the HA-tagged constitutively active Akt (CA-Akt) before transfection with a high dose of 3p-RNA in CAL27 cells. As shown in Figure 7B, the cleavage of PARP induced by high-dose 3p-RNA was abrogated by CA-Akt. Consistently, the interaction of caspase-9 with RIG-I was also blocked by CA-Akt (Fig. 7C). These results demonstrate that the apoptosis induced by high-dose 3p-RNA is dependent on decreased activation of Akt.

## Discussion

Activation of PRR leads to production of inﬂammatory cytokines, including TNF-α, IL-6 and IFN-β, which are essential for both innate and adaptive immunity to clear pathogens [10], [20]. Our results here show that as a PRR that functions in innate immunity against virus infections, RIG-I is also highly expressed on HNSCC cells and its activation contributes to tumor growth and progression in response to a low dose of 3p-RNA, whereas leading to apoptosis in response to a high dose of 3p-RNA.

RIG-I is localized in the cytosol and recognizes 3p-RNA generated by viral RNA polymerases in the cytosol of cells. 3p-RNA is also generated physiologically in the nucleus, but it lose its RIG-I ligand activity before it is released to the cytosol due to processing such as capping of the 5′-triphosphate end or nucleoside modification of RNA [6], [7]. Active 3p-RNA can be generated by *in vitro* transcription and used as synthetic ligands for RIG-I [11]. 3p-RNA shares certain structural features with siRNAs, and the requirements for cytosolic delivery are similar. Several siRNA molecules are in clinical studies, and short 3p-RNA may benefit from formulations developed for the clinical application of siRNAs [11].

The successful survival of a virus relies upon the evolution of strategies that modulate host cell signaling pathways, particularly those governing apoptosis and cell survival [21]. Infected host cells undergo apoptosis to eliminate invading viruses, but virus-encoded anti-apoptotic proteins can also counter this response to allow completion of the virus replication cycle before destruction occurs [22]. Activation of PI3K/Akt signaling is believed to contribute to the maintenance of the latent state by suppressing apoptosis and hence the elimination of virus-infected cells [23]. PI3K/Akt signaling promotes cell survival by multiple mechanisms, such as phosphorylating and inactivating caspase-9 or the BH3 domain containing family member BAD, or activation of PRL-3 [24]. Viral products, such as LMP1 of Epstein Barr Virus (EBV), can activate PI3K either through direct interaction with the catalytic or adaptor subunits or by facilitating the association of PI3K with receptor or non-receptor tyrosine kinases [25], [26]. Our results show that activation of the PI3K/Akt and NF-κB pathways by transfection with the double-stranded RNA virus mimic 3p-RNA enhances cellular proliferation and functions to benefit virus survival, suggesting that virus dsRNA can also promote host cell survival.

Apoptosis is a common mechanism used by the host to block viral spread. For example, virus-induced apoptosis of peripheral neuronal cells can be a protective host response that blocks transmission of herpes simplex virus 2 (HSV-2) to the central nervous system [27]. However, late in infection, apoptotic death of the infected cell may facilitate virus egress and spread of the infection [28]. Thus, the timing of apoptosis is a critical factor in determining whether it is pro- or anti-viral. Our results showing that transfection of low-dose 3p-RNA led to proliferation of infected cells may represent the early infection stage, during which the virus promotes host cell survival. Conversely, the induction of apoptosis by a high dose of transfected 3p-RNA may represent the late infection stage, during which the virus requires the cell lytic process to spread. Our finding that viral dsRNA also functions in the progression of infection gives new insight for future molecular research on virus infections.

Hanahan and Weinberg suggested essential alterations in cell physiology that collectively dictate malignant growth, and evasion of apoptosis or resisting cell death is an important hallmark of tumor cells [2]. Based on growing understanding of the molecular mechanisms underlying apoptosis resistance to tumors, many studies have attempted to induce tumor cell apoptosis by targeting anti-apoptotic proteins [29], [30]. We found that low dose of 3p-RNA lead to cell proliferation by activating NF-κB signal pathway, which suggests that virus infection-activated RIG-I is an oncogenic factor at early stage of HNSCC initiation. This viewpoint adds new explanation of how HPV infection frequently leads to head and neck cancers [31]. This work demonstrated that RIG-I activation could lead to apoptosis by interaction with caspase-9 in CAL27 cells, in which Akt activation was shown to be down-regulated. Additionally, overexpression of constitutively active Akt could inhibit the interaction of RIG-I and caspase-9, indicating that activation of Akt mediates dsRNA-induced apoptosis. We now speculate that the structure of two CARD domains containing RIG-I favoring interacting with caspase-9 upon high-dose 3p-RNA transfection, which is different from that upon low dose of 3p-RNA or common dose of virus infection. Why transfection of high-dose 3p-RNA does not induce Akt activation with upregulation of Akt inhibitor phosphatase PTEN or PP2A will require further investigation [32]. 3p-RNA delivered by PEI *in vivo* has been shown to induce apoptosis of melanoma tumor cells and strongly activate NK cells at the same time [33]. Our results suggest that 3p-RNA delivered *in vivo* may also yield promising results in the HNSCC model.

In conclusion, we identified a signaling pathway which results in either proliferation or apoptosis, depending upon the dose of 3p-RNA, and described an additional physiological function for viral dsRNA in the HNSCC model. Our findings support that effective *in vivo* delivery of potent synthetic RNA ligands may be a promising approach for the treatment of HNSCC.

## Supporting Information

Figure S1 **SCC9 or SCC25 cells (5×10^5^ per well) were plated in 12-well plates overnight and then stimulated with PBS or LPS for 12 h or transfected with 100 ng/ml 3p-RNA for 16 h.** mRNA expression levels of RIG-I and MDA-5 were analyzed by Q-PCR. The basal level of MDA-5 normalized by β-actin was used as the control.
(TIF)

Figure S2 **SCC4 cells treated as in** **Figure 4A** **were collected for migration and invasion assays.** Results are presented as fold increases over the basal level of the control. The numbers of cells in the membranes were analyzed after 24 h. MTT results are presented as the absorption ratio. Similar results were obtained in triple repetitions of experiments, *P<0.01, Student’s t-test).
(TIF)

Figure S3 **SCC4 cells were transfected as in** **Figure 5A** **and then stained with Annexin V and propidium iodide.** Apoptotic cells are indicated as the percentage of gated cells.
(TIF)
