# Supplementary figures and images for: Dose Dependent Activation of Retinoic Acid-Inducible Gene-I Promotes Both Proliferation and Apoptosis Signals in Human Head and Neck Squamous Cell Carcinoma
Source: PLoS One. 2013 Mar 4;8(3):e58273. doi: 10.1371/journal.pone.0058273 (PMC3587586; doi:10.1371/journal.pone.0058273)

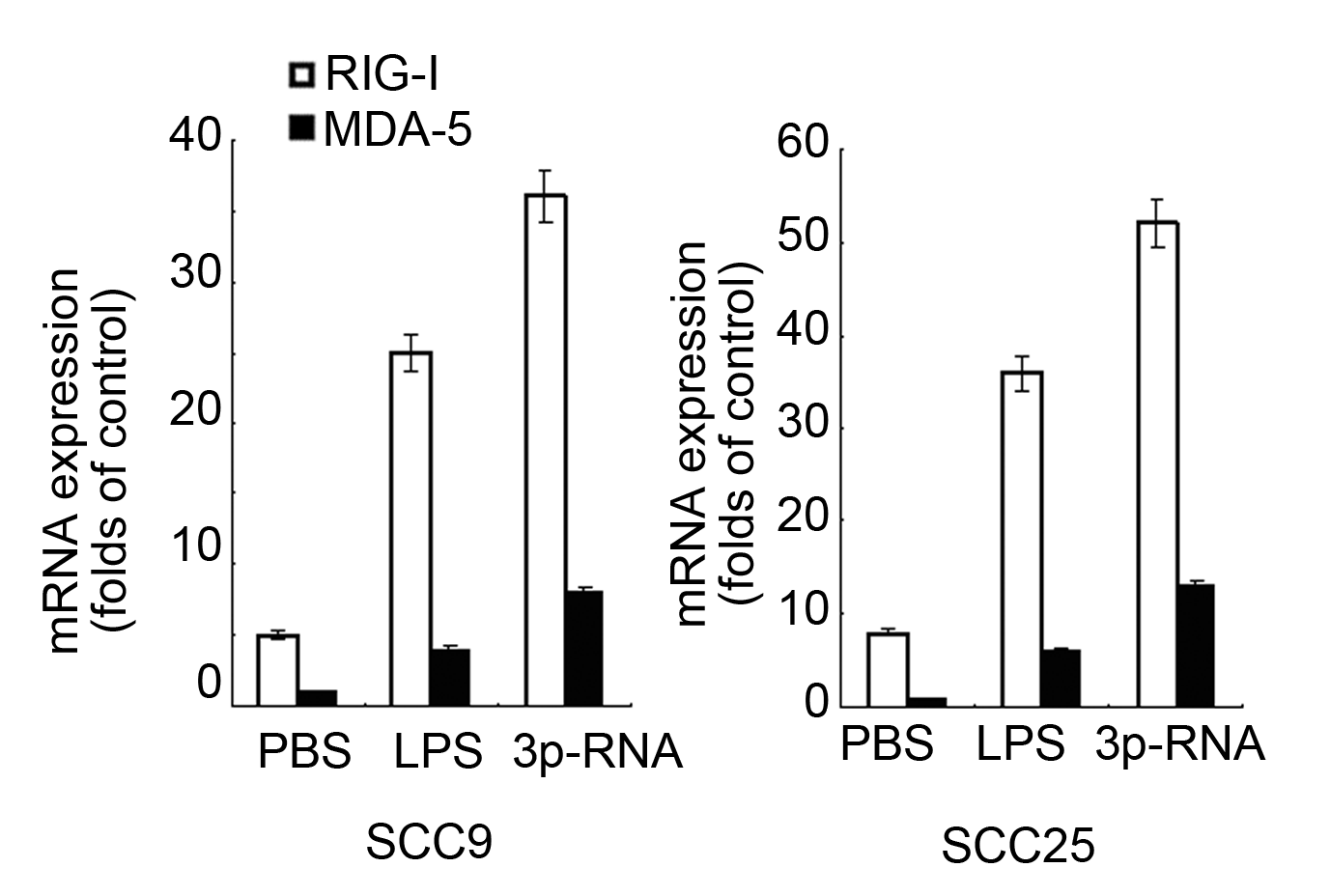

Supplement: Figure S1 — SCC9 or SCC25 cells (5×10^5^ per well) were plated in 12-well plates overnight and then stimulated with PBS or LPS for 12 h or transfected with 100 ng/ml 3p-RNA for 16 h. mRNA expression levels of RIG-I and MDA-5 were analyzed by Q-PCR. The basal level of MDA-5 normalized by β-actin was used as the control. (TIF) [file pone.0058273.s001.tif]

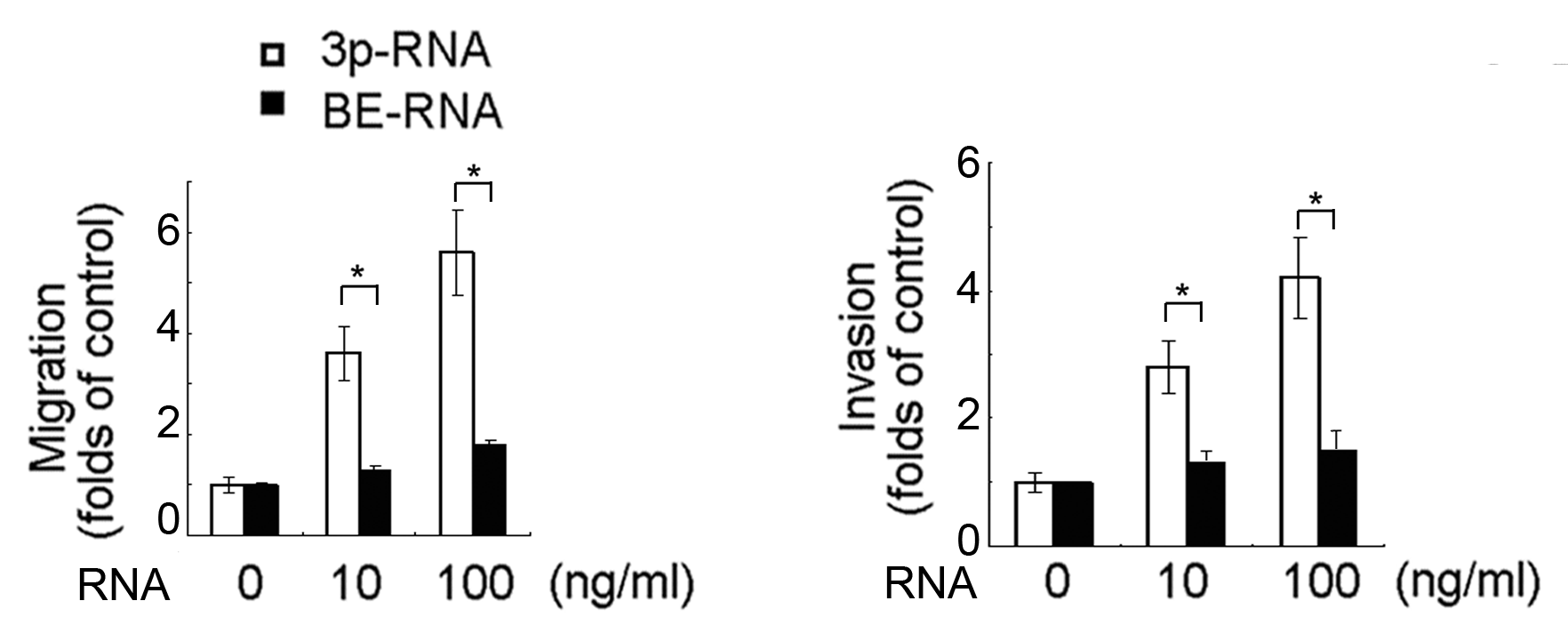

Supplement: Figure S2 — SCC4 cells treated as in Figure 4A were collected for migration and invasion assays. Results are presented as fold increases over the basal level of the control. The numbers of cells in the membranes were analyzed after 24 h. MTT results are presented as the absorption ratio. Similar results were obtained in triple repetitions of experiments, *P<0.01, Student’s t-test). (TIF) [file pone.0058273.s002.tif]
